# Collecting Protein Biomarkers in Breath Using Electret Filters: A Preliminary Method on New Technical Model and Human Study

**DOI:** 10.1371/journal.pone.0150481

**Published:** 2016-03-02

**Authors:** Wang Li, Xitian Pi, Panpan Qiao, Hongying Liu

**Affiliations:** 1 Key Laboratory of Biorheology Science and Technology, Ministry of Education, College of Bioengineering, Chongqing University, Chongqing, PR China; 2 Key Laboratories for National Defense Science and Technology of Innovative Micro-Nano Devices and System Technology, Chongqing University, Chongqing, PR China; 3 Chongqing Engineering Research Center of Medical Electronics, Chongqing, PR China; Kermanshah University of Medical Sciences, ISLAMIC REPUBLIC OF IRAN

## Abstract

Biomarkers in exhaled breath are useful for respiratory disease diagnosis in human volunteers. Conventional methods that collect non-volatile biomarkers, however, necessitate an extensive dilution and sanitation processes that lowers collection efficiencies and convenience of use. Electret filter emerged in recent decade to collect virus biomarkers in exhaled breath given its simplicity and effectiveness. To investigate the capability of electret filters to collect protein biomarkers, a model that consists of an atomizer that produces protein aerosol and an electret filter that collects albumin and carcinoembryonic antigen-a typical biomarker in lung cancer development- from the atomizer is developed. A device using electret filter as the collecting medium is designed to collect human albumin from exhaled breath of 6 volunteers. Comparison of the collecting ability between the electret filter method and other 2 reported methods is finally performed based on the amounts of albumin collected from human exhaled breath. In conclusion, a decreasing collection efficiency ranging from 17.6% to 2.3% for atomized albumin aerosol and 42% to 12.5% for atomized carcinoembryonic antigen particles is found; moreover, an optimum volume of sampling human exhaled breath ranging from 100 L to 200 L is also observed; finally, the self-designed collecting device shows a significantly better performance in collecting albumin from human exhaled breath than the exhaled breath condensate method (p<0.05) but is not significantly more effective than reported 3-stage impactor method (p>0.05). In summary, electret filters are potential in collecting non-volatile biomarkers in human exhaled breath not only because it was simpler, cheaper and easier to use than traditional methods but also for its better collecting performance.

## Introduction

Though volatile organic compounds (VOCs) in human breath have been studied and reviewed in depth in recent decades [1–10], increasing interests are seen in studies on non-volatile biomarkers in exhaled breath of human volunteers. Biomarker collection approaches have been abundant yet at different collection efficiencies [11, 12]. Among them is the exhaled breath condensate (EBC) method, where biomarkers, in the form of epithelial lining fluid (ELF) droplets from airway [13], are diluted approximately 20000-fold by condensed water vapor [14]. Unfortunately, the resulting concentration of biomarkers often falls below the detection limit of commercially available equipment, let alone the repeatability and verifiability [15–17]. It has been reported that inner coating of collection surface could make EBC method more efficiency, however, different coatings or even different condensing systems favor different biomarkers in breath [18]. Moreover, this method offers limited help in *in vivo* study [18]. The reasons include that the EBC method wastes more than 90% of the sub-micron particles in exhaled breath [19].

3-stage impactor is the second avenue to collect particles in exhaled breath based on inertia [20]. In particular, particles with greater inertia attach to the plate in the first stage, while those with less inertia flow through nozzles and enter into the following stages. Although such method have been reported to successfully collect protein particles [21, 22], the complexity of collection device and plate sanitation inhibit its widespread use.

The third and most recent means is electret filter that collects exhaled particles through electrostatic forces, Brownian diffusion, inertial impaction, and interception. The former two work on sub-micron particles (<1 μm) while the rest target bigger particles [11]. In human exhaled breath, most particles are charged (as their isoelectric points don’t equal to the pH of exhaled breath) or polarized by the electret filter with dimensions on the order of sub-microns [23–25], and thus allow for collection via electret filters using electrostatic forces. Collection of biomarkers such as influenza virus and human rhinovirus using electret filters has been attempted with success [26, 27], but studies on the collection of protein biomarkers using this method are rarely seen. One study compares cytokine particle collection using electret filter to that using a SKC Biosampler^®^ and Omni 300^TM^ but without consideration of collection capability changes (especially in samples contain liquid particles such as human exhaled breath) of the electret filter [11].

In this paper, a model that combines production and collection of liquid protein particles using electret filters is developed. Carcinoembryonic antigen (CEA)-a common biomarkers in lung cancer development [28, 29]and potential biomarker in early detection of lung cancer-from atomized aerosol and albumin-the indicative of early asthma deterioration [30]and a widely used reference marker of dilution in bronchoalveolar lavage fluid [31, 32]-both in atomized particles and human exhaled breath are studied. To our best knowledge, collection of these two proteins in either atomized aerosol or human exhaled breath is not reported.

A self-designed collection device using electret filter as collection medium is compared to two previously reported collecting methods in terms of the amounts of albumin in human exhaled breath collected. The results shows that electret filter has a better performance than EBC method though it is not more effective than 3-stage impactor method in collecting protein particles from human exhaled breath.

## Materials and Methods

### Model experiment

#### Experiment setup

**Fig 1:**
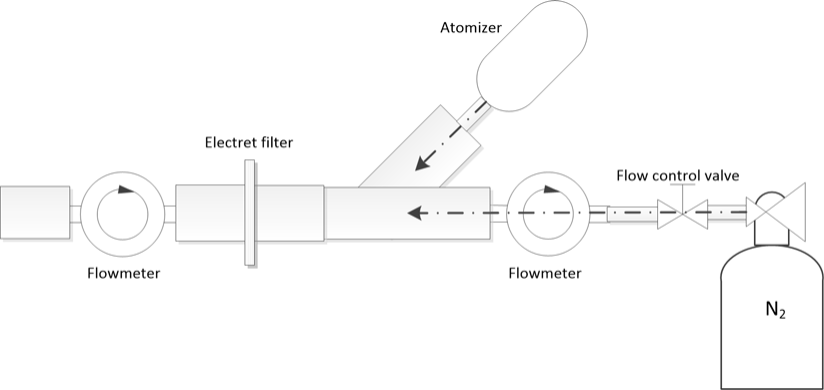
Schematic diagram of experiment setup used in this study.

A model was set up to produce aerosol particles containing proteins to mimic the “exhalation” scenario (Fig 1). The model used a medical atomizer (FOLEE, China) to produce aerosol particles and high purity nitrogen to carry the particles to the collection site.

Electret filters (North, Honeywell Inc., Morristown, NJ) with the same lot number were cut into circular shapes with diameters of 2 cm. When collecting, the circular electret filters were fixed in the collection site of the pipe (Fig 1). To make the atomization solutions, CEA and albumin (Cloud-Clone Corp., Houston, TX) were dissolved in phosphate buffer saline (PBS, 0.01 M and pH 7.4). The final concentrations of CEA and albumin were 2.5 ng/ml and 50 ng/ml, respectively. The protein solutions were then atomized by the atomizer to produce aerosol particles in diameters ranging from 0.5μm to 10μm. The produced particles were sprayed into the collection pipe by the atomizer at a flow rate of 5 L/min. Then the high purity nitrogen delivered the aerosol particles to the electret filter in the collection site. Flow rate of the carrier gas was kept at 10 L/min by a control valve and was monitored by a gas flowmeter (Siargo Inc., Santa Clara, CA). The overall flow rate was maintained at 15 L/min which was measured using gas flow meter (Siargo Inc., Santa Clara, CA).

#### Study method

To check atomizer stability during the experiment, the atomizing rate was firstly assessed by measuring amounts of atomized PBS (0.01 M, pH 7.4) solution in different atomizing durations. In the model experiment, 7 circular electret filters were then used to collect aerosol particles atomized using prepared CEA solution for 7 different periods (collecting particles for 1 min, 2 min, …, 7 min, respectively) according to the collection method introduced above. Collecting albumin particles produced by atomizing the albumin solution was also performed to validate the collection effectiveness of the electret filter for protein particles. In the control group, only PBS (0.01 M, pH 7.4) solution containing no protein was used instead of protein solutions to produce aerosol particles. Other treatments in the control group were the same as in the normal group.

The weights of the filters and the atomizing cup of the atomizer containing the protein solution were measured before and after collection with a high precision electronic scale (Sartorius AG, Gottingen, Germany).

### Collecting human exhaled breath particles

#### Device design

**Fig 2:**
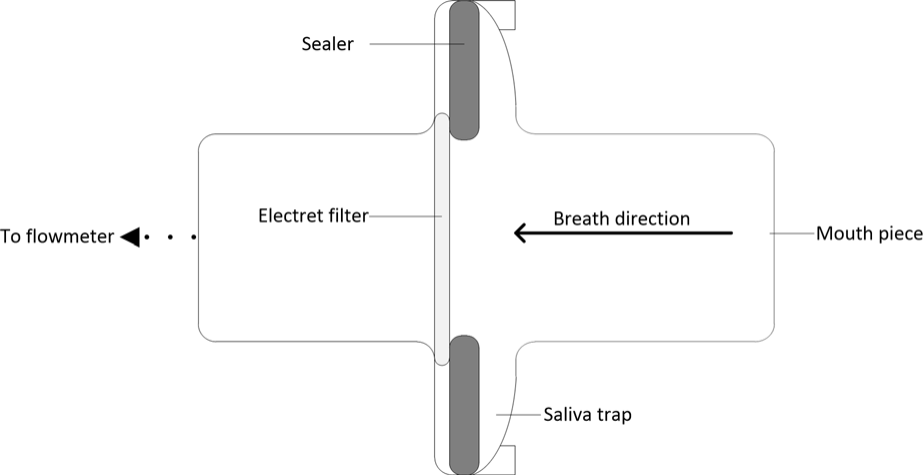
Schematic diagram of the designed collecting device in vertical section.

Based on the effectiveness of collecting protein in atomized particles, collecting protein in human exhaled breath particles was also studied. A device simply consisted of a mouth piece, a saliva trap and a rubber sealer was designed for this purpose (Fig 2). The electret filter (diameter 2 cm) was also used as the collecting medium fixed in the collecting site of the device. When collecting, a gas flow meter (Siargo Inc., Santa Clara, CA) was connected at the outlet end of the device to measure the volume of exhaled breath.

#### Study population

Six healthy volunteers recruited from Chongqing, China, participated in this study. Three men (S-2 and S-5 were smokers with no clinical overt disease) and three women were involved. Protocols of this study was approved by Medical Ethics Committee of Chongqing. All volunteers signed the informed consent after the procedure of the study was explained in detail. Additional information about the volunteers is provided in Table 1.

**Table 1:** Study population.

M/F^a^	Age (years) Median (range)	Smokers
3/3	35 (24,54)	2/6

^a^M/F: number of males and females

#### Study design

**Fig 3:**
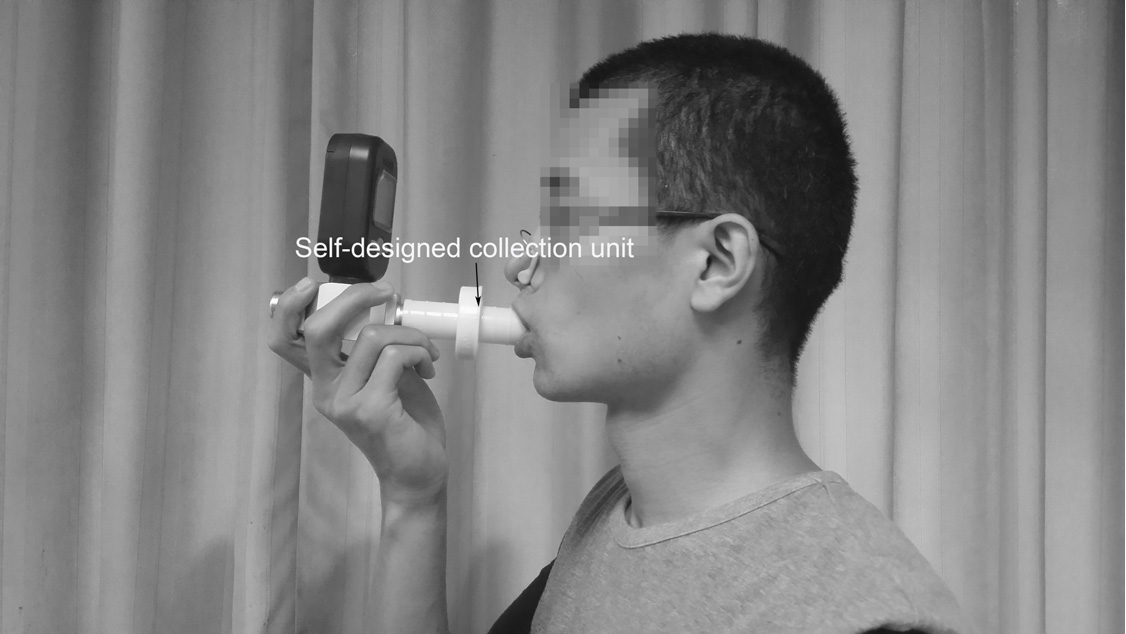
Collecting exhaled breath particles using a self-designed collecting device.

According to previous reports, particle concentration increases 10-70-folds in exhaled breath after taking a deep breath and exhaling to residual volume comparing to tidal breathing [25]. Thus a similar breathing maneuver was employed in this experiment. All volunteers were asked to wear a nose clip throughout the collecting procedure (Fig 3). The detailed breathing maneuver was described as follows:

1. Before collecting, all volunteers rinsed their mouth for 3 minutes with purified water and breathed deeply for 1 minute.
2. Volunteers inhaled the ambient air to their vital capacities.
3. Volunteers exhaled the breath into the designed collection device horizontally at a rate of 50 L/min until reaching their residual volumes.
4. Volunteers repeat step 2 and step 3 to give desired volumes of breath.

During the course of collection, volunteers were asked to swallow their accumulated saliva to avoid contamination. Particles from 7 different breath volumes (50 L breath, 100 L breath, …, 350 L breath, respectively) obtained from each volunteer were collected. In the control group, 7 designed collecting devices were implanted to the inlet port of an air pump that intake ambient air at the flow rate of 50 L/min. Volumes of air passed though the filters were the same as the volumes of human exhaled breath.

Because albumin is widely used as a reference marker of dilution in bronchoalveolar lavage fluid [31, 32], and thus indicates albumin is relatively constant in the epithelial lining fluid (ELF) of healthy volunteers. Only the concentration of exhaled albumin was measured for its relatively stable origin.

### Filter treatment after collecting

After collecting particles, all filters were cut into pieces and transferred to 5 ml centrifuge tubes (Eppendorf, Hamburg, Germany). 2 ml of eluent (0.01 M PBS containing 0.13% Tween-20) was added to the tubes. Filter pieces were flushed repeatedly using the eluent until all pieces were fully wet. The tubes containing filter pieces were then transferred into an ultrasonic cleaner (cleaning for 5 minutes) to wash out proteins collected by the filters. After that, 1 ml of the eluate was transferred to a 1.5 ml centrifuge tube (Eppendorf, Hamburg, Germany) and centrifuged under 1000 r/min for 2 minutes (Centrifuge 5804R, Eppendorf, Germany). At last, 0.5 ml supernatant of the eluate was used to measure protein concentrations.

### Protein concentration measurement

Salivary amylase was firstly measured to ensure that samples were not contaminated by saliva. Concentrations of CEA and albumin in the samples were measured using commercial SEAl50Hu and HEB028Hu ELISA kit (Cloud-Clone Corp., Houston, TX) respectively according to the manufacturer’s instructions and analyzed using the same ELISA plate. A microplate reader (Tecan, Mannedorf, Switzerland) was used to read plate absorbance at 450 nm (the reference wavelength was set at 620 nm). Protein concentrations were calculated from a four-parametric standard curve fitted using OriginPro V9.0.0 (OriginLab Corporation, Northampton, MA).

### Calculations

Generally, weights of electret filters increased after collection. We calculated the weight increase of the filter as follows:

$$\Delta m=m_{FB}\;\text{-}\;m_{FA}$$ (1)

where *Δm* (g) is weight increase of an electret filter, *m*_*FB*_ (g) is the weight of the filter before collecting, and *m*_*FA*_ (g) is the weight of the filter after collecting.

Because protein concentrations in a nebulizer solution (and in exhaled breath of human) were very low, the weight increase of the filter after collecting was mainly caused by water. The amount of protein collected using an electret filter can be calculated as follows:

$$M_{C}=(V_{E}+\frac{\Delta m}{\rho })C_{C}$$ (2)

where *M*_*C*_ (ng or pg) is the amount of protein collected using the electret filter, *Δm* (g) is the amount of liquid particles that the filter collected, *C*_*C*_ (ng/ml or pg/ml) is the protein concentration measured by ELISA (*C*_*C*_ below the limit of detection was assigned equal to *LOD* / √2) [22], *ρ* (1 g/ml) is the density of water, and *V*_*E*_ (2 ml) is the volume of eluent used.

Mean collecting efficiencies of protein aerosol particles using an electret filter were calculated as follows:

$$E_{C}=\frac{M_{C}}{C_{pB}V_{cB}-C_{pA}V_{cA}}•100\%$$ (3)

where *E*_*C*_ (%) is the collecting efficiency of the electret filter method, *C*_*pB*_ and *C*_*pA*_ (both in pg/ml or ng/ml) are the concentrations of proteins in the nebulizer measured using ELISA before and after atomization, respectively. And *V*_*cB*_ (ml) and *V*_*cA*_ (ml) are the volumes of the protein solution in the nebulizer cup before and after atomization respectively. These volumes were calculated by weight changes of the protein solutions in the atomization cup.

### Statistics

In the model experiment, Levene’s test was firstly used to assess the equality of variances of the obtained data if the variances in the population differed significantly (p<0.05), and Kruskal-Wallis ANOVA was performed if the amount of atomized protein solution significantly influenced the weight increase of the filters and the amounts of proteins collected.

In the experiment collecting human exhaled breath particles, Kruskal-Wallis ANOVA was also performed to analyze whether breath volumes of each volunteer significantly affected the weight increase of the filter and the amount of albumin collected. Finally, Kruskal-Wallis ANOVA was performed again to test if the collecting efficiencies of 3 different methods were significantly different from each other.

All statistical analyses were performed using Origin Pro 9.0.0 (OriginLab Corporation, MA, USA).

## Results

The mean percentage change of the atomizing rate for different collecting durations was 3.85%±2.88% (mean±SD). This little change of the atomizing rate would guarantee a stable origin of aerosol particles for collection experiment.

**Fig 4:**
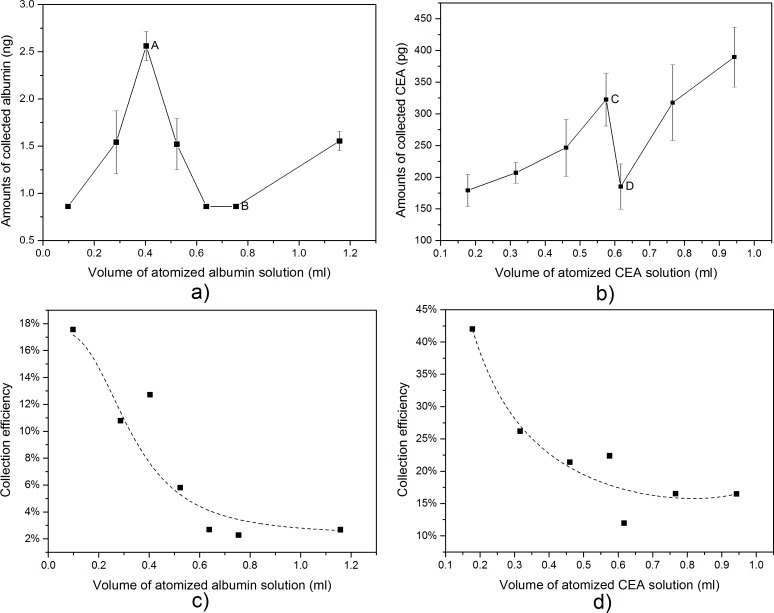
Collecting ability of electret filters in collecting atomized protein particles. (a) Amounts of collected albumin when the volume of atomized albumin solution increased. Amounts of collected albumin increased as the volume of atomized albumin solution increased before point A, then decreased, and finally increased again after point B. (b) Amounts of collected CEA when the volume of atomized CEA solution increased. Amounts of collected CEA increased as the volume of atomized CEA solution increased before point C, then decreased, and finally increased again after point D. (c) Mean collecting efficiency of electret filters when the volume of atomized albumin solution increased. (d) Mean collecting efficiency of electret filters when the volume of atomized CEA solution increased. Amounts of collected proteins were calculated by Eq 2. Collecting efficiencies were calculated by Eq 3. Error bars shown in a) and b) were standard deviation of triplicate experiments.

In the model experiment, the amounts of collected proteins (both CEA and albumin) in the atomized particles had a similar trend with the volume of the atomized protein solution increased (Fig 4). At first, the amounts of collected protein increased with the volume of atomized protein solution, then deceased, and finally increased again. Nevertheless, our results showed that the volume of atomized protein solution had no significant effect on the amounts of collected protein (Kruskal-Wallis ANOVA, p>0.05). In addition, decreased collecting efficiencies of the electret filters in this experiment were observed. As shown in Fig 4, a decrease of collection efficiency for atomized albumin particles from 17.6% to 2.3% and a decrease of collection efficiency for atomized CEA particles from 42% to 12.5% were found.

**Fig 5:**
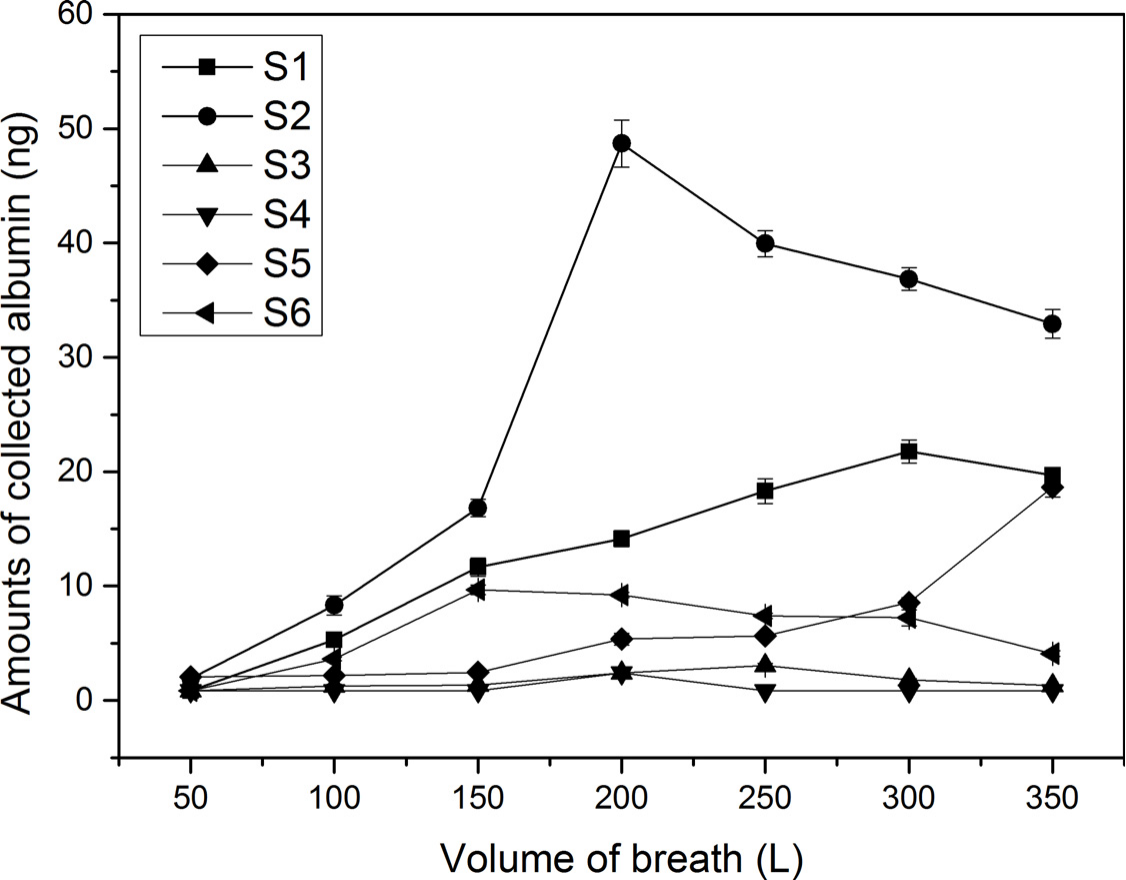
Amounts of albumin in human exhaled breath collected using self-designed collecting devices. The amounts of collected albumin were calculated by Eq 2. Error bars shown in the figure were standard deviation of triplicate measurements of each sample.

In the experiment collecting human exhaled breath particles, amounts of collected albumin had a similar trend as the volume of breath increased comparing to the model experiment (except volunteer S-5). However, amounts of collected albumin did not increase in the final stage when comparing to the model experiment (Fig 5). In addition, different volunteers and different breath volumes of the same volunteer significantly affected the collected albumin (Kruskal-Wallis ANOVA, p<0.05). However, an optimum breath volume range of 100 L-200 L for collecting human exhaled breath particles using the electret filter method can be determined from the obtained data. A smaller breath volume may not be able to collect enough target biomarkers to be detected, and a higher breath volume may face the same problem and, at the same time, increase the discomfort of volunteers.

In both experiments, the weight of all of the electret filters increased after collection (Table A and Table B in S1 File). In the model experiment, increased weights of the filters were not significantly affected by the volume of atomized protein solution (Kruskal-Wallis ANOVA, p>0.05). However, exhaled breath volume was found significantly affected the weight increase of filters when collecting human exhaled breath particles (Kruskal-Wallis ANOVA, p<0.05).

**Fig 6:**
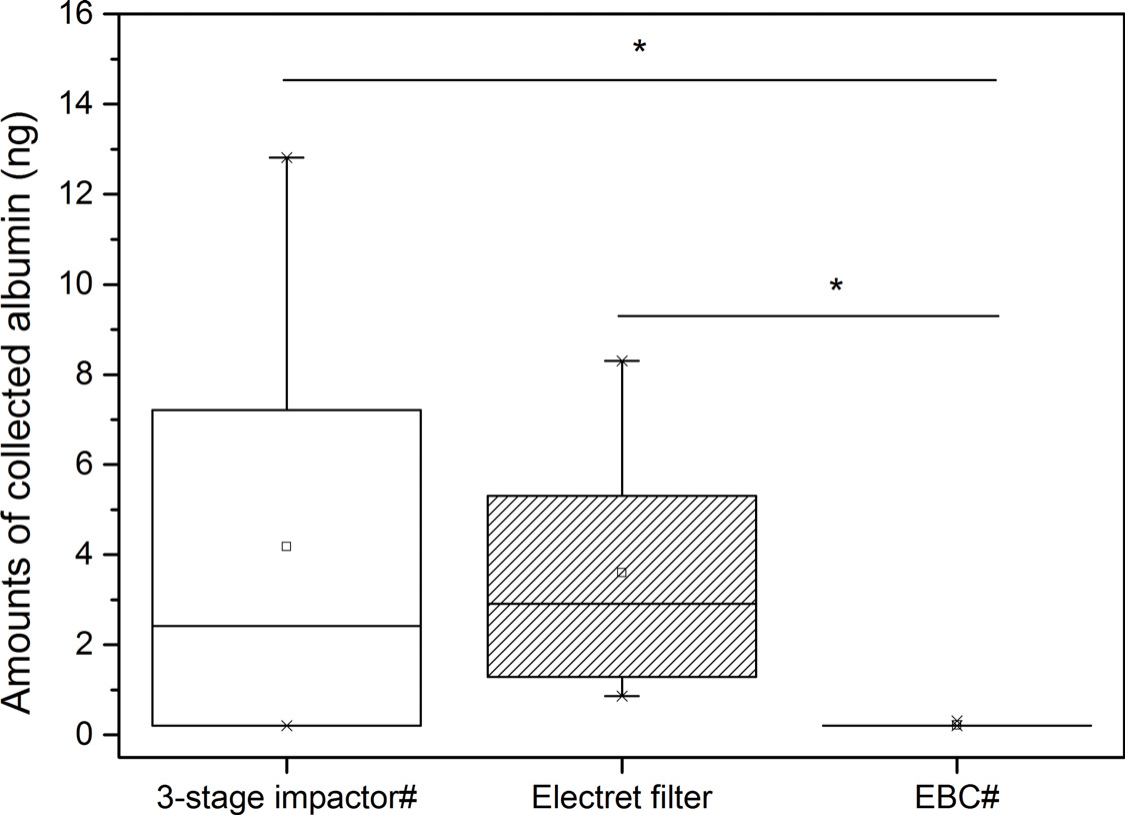
Comparison of collecting ability of 3 different collecting methods. *indicates significance at p<0.05. # indicates boxplot was drawn by the author from data in [22].

A comparison of collecting ability of the electret filter method with other 2 reported methods (3-stage compactor and EBC method) was performed based on the amounts of albumin collected from exhaled breath particles (Fig 6). The breathing maneuvers of volunteers and the volume of exhaled breath were the same as in the 2 reported methods. The results showed that our self-designed collection device based on the electret filter method was significantly more effective than the EBC method in collecting albumin from human exhaled breath (Kruskal-Wallis ANOVA, p<0.05) but was not more effective than the 3-stage impactor (Kruskal-Wallis ANOVA, p>0.05).

In the model experiment, electret filters that collected particles produced using PBS contained no detectable levels of albumin and CEA. In the experiment collecting human exhaled breath particles, electret filters that collected particles in ambient air contained no detectable levels of albumin.

## Discussion

Increases and subsequent decreases in the effectiveness of collecting protein using an electret filter in both the model and human volunteer experiments were observed in this study. The reasons were listed as follows:

1. At the beginning of collection, sub-micron particles (<1μm) in atomized aerosol (or in exhaled breath) were collected because of electrostatic forces between the particles and the electret fiber [11, 23]. Therefore, collected proteins increased as the volume of atomized protein solution (or exhaled breath) increased. Increased protein concentration in the atomized droplets may also play a role in increasing the amounts of collected protein in the model experiment [33].
2. As the collecting procedure progressed, more and more liquid particles were collected by the electret fiber, and a water layer was formed around the fiber. This layer can weaken the electric field around the fibers [34]. On the other hand, the opposite particle charge of the particles can also neutralize the electret charge to decay the electrostatic forces [34]. Both factors contributed to the subsequent decrease of collecting effectiveness of the electret filters.

In the model experiment, amounts of collected protein both increased after point B and point D in the collection curve (Fig 4). However, a similar trend was not observed in the experiment of human volunteers (Fig 5). The difference may be partly caused by different particle size distribution in these two experiments. The atomized aerosol contained more large particles (>0.5μm) while exhaled breath contained fewer [24, 25, 35]. Because the electret fibers were covered by a water layer, large particles were more easily captured as a result of inertial impaction. An increasing concentration of protein particles in atomized droplets may also have contributed to the increase of collected protein in the model experiment [33].

As discussed above, the electrostatic forces of the electret filter were weakened during the collection of liquid particles. As introduced earlier, electrostatic forces were main mechanisms of electret filters in collecting sub-micron particles (<1μm), thus a decay in electrostatic forces caused a decrease of collection efficiency of the electret filter, as seen in Fig 4C and Fig 4D. This finding agreed with the results obtained by Ji *et al* who collected atomized NaCl and dioctyl sebacate particles using electret filters [36]. These results may indicate that, in future experiments, an effective method for obtaining relatively higher concentrations of collected protein in exhaled breath particles is to dry the exhaled breath or use more non-wettable electret filters.

Contrary to the results obtained from human volunteers, the weight of the electret filters and the amounts of collected protein were not significantly affected by an increase of the volume of the atomized solution. This difference may be also caused by bigger liquid particles in our model experiment, in which inertial impaction played a role. Nevertheless, weight changes (mainly caused by water) of the electret filter in both the model and human volunteer experiments were negligible (<0.015 g) when comparing to 2 ml of eluent added in the subsequent experiment.

Rosias *et al* reported EBC method obtained 69.4% of albumin recovery in *in vitro* study and 7/13 albumin positive detection in *in vivo* study (asthmatic children as volunteers) using silicone coating in the collection surface [18]. However, the electret filters method showed a better performance. The albumin recovery rate in our model experiment before elution process reached more than 2500% (though the final albumin recovery rate drop to less than 2.5% because of more than 1000-folds dilution in subsequent elution process in the study, data calculated based on Table B in S2 File). And our human study (healthy adults as volunteers) showed 6/6 of albumin positive detection. The results indicated that electret filter method had a better collection ability than the EBC method in collecting albumin in exhaled breath. It was consistent with comparison results demonstrated in Fig 6.

Even so, EBC method may collect some volatile biomarkers (such as NH_4_^+^ and HCO_3_^-^ in condensate) that electret filters cannot collect effectively. Although our self-designed collecting device was not more effective than the 3-stage impactor in collecting albumin in human breath particles, it was much cheaper, simpler and easier to use. The device can be even used as a disposable collection accessory to collect particle biomarkers when using other respiratory equipment (e.g., a spirometer). In addition, the electret filter collecting method has the potential to obtain higher protein concentrations by, for example, drying the breath samples, using more non-wettable electret filters and reducing consumption of eluent. Moreover, charging the exhaled breath particles may make the electret filter more effective in collecting them [37, 38].

### Limitations of study

Firstly, we did not study the collecting efficiency of electret filters in different particle concentrations. Different concentrations of the liquid particles may decay the electrostatic force of the electret filter differently and thus influence their collecting efficiency. The fact is, to our knowledge, particle concentration in different people varies significantly [39, 40].

Secondly, we did not consider the charging state of the particles (both in atomizer and exhaled breath). Actually, according to the formula obtained by Chikao Kanaoka *et al* [41], the collecting efficiency of electret filter would change as the charging state of the particles. To address this problem, exhaled samples should be charged or uncharged uniformly in future studies.

Moreover, electret filters can only collect non-volatile biomarkers in aerosol particles of breath, volatile biomarkers (volatile organic compounds, for example) in exhaled breath cannot be collected by this method.

Finally, only 6 volunteers and two model proteins were included in our study. To obtain more conclusive results, more volunteers and biomarkers should be involved.

## Conclusions

Our study found successful collection of proteins in both atomized aerosol and exhaled breath particles using electret filters, though with a decreasing collection efficiency. In addition, the amounts of collected albumin in exhaled breath vary with volunteers and the volume of exhaled breath. Furthermore, an optimum breath volume ranging from 100L to 200 L for collecting proteins in exhaled breath particles using the electret filter method was found.

We also found that our self-designed collecting device using an electret filter as the collecting medium had a much better collection ability than traditional EBC method in collecting non-volatile biomarkers in exhaled breath. Although it was not more effective in collecting albumin in human exhaled breath particles than the 3-stage impactor, it was much simpler, cheaper and easier to use, and had more potential to improve its performance. However, volatile biomarkers in exhaled breath cannot be collected by this method. Future research includes assessing the performance of collecting other particle biomarkers in exhaled breath using this method and improving the collection performance of the electret filter method by appropriate desiccation of exhaled breath sample, using more non-wettable electret filters and a better eluting method which could considerably reduce usage of eluent and charging biomarker particles in the sample uniformly.

## Supporting Information

S1 File Weight of electret filters before and after collecting proteins in model and human experiment. (DOCX)

S2 File Other raw data of collecting proteins in model and human experiment. (XLSX)
