# Supplementary material for: Collecting Protein Biomarkers in Breath Using Electret Filters: A Preliminary Method on New Technical Model and Human Study
Source: PLoS One. 2016 Mar 2;11(3):e0150481. doi: 10.1371/journal.pone.0150481 (PMC4775061; doi:10.1371/journal.pone.0150481)
Supplement: S1 File — (DOCX) [file pone.0150481.s001.docx]

| Table A． Weights of the electret filters before and after collecting atomized albumin, CEA and PBS solutions | **PBS (0.01M, PH7.4)** | **Δm(g)** | **0.001** | **0** | **0** | **0.002** | **0.001** | **0.002** | **0** |
| --- | --- | --- | --- | --- | --- | --- | --- | --- | --- |
|  |  | **After(g)** | 0.197 | 0.295 | 0.339 | 0.323 | 0.321 | 0.316 | 0.305 |
|  |  | **Before(g)** | 0.196 | 0.295 | 0.339 | 0.321 | 0.32 | 0.314 | 0.305 |
|  |  | **Atomized amount(g)** | 0.138 | 0.304 | 0.385 | 0.45 | 0.613 | 0.864 | 0.963 |
|  |  |  |  |  |  |  |  |  |  |
|  | **CEA solution (2.5ng/ml)** | **Δm(g)** | **0.001** | **0.001** | **0.003** | **0.004** | **0.007** | **0.005** | **0.005** |
|  |  | **After(g)** | 0.322 | 0.291 | 0.257 | 0.306 | 0.317 | 0.297 | 0.392 |
|  |  | **Before(g)** | 0.321 | 0.29 | 0.254 | 0.302 | 0.31 | 0.292 | 0.387 |
|  |  | **Atomized amount(g)** | 0.168 | 0.307 | 0.51 | 0.592 | 0.637 | 0.786 | 0.964 |
|  |  |  |  |  |  |  |  |  |  |
|  | **Albumin solution (50ng/ml)** | **Δm(g)** | **0.002** | **0.001** | **0** | **0.001** | **0.001** | **0.002** | **0.002** |
|  |  | **After(g)** | 0.299 | 0.288 | 0.308 | 0.297 | 0.301 | 0.356 | 0.35 |
|  |  | **Before(g)** | 0.297 | 0.287 | 0.308 | 0.296 | 0.3 | 0.354 | 0.348 |
|  |  | **Atomized amount(g)** | 0.181 | 0.256 | 0.513 | 0.58 | 0.741 | 0.811 | 1.107 |
|  |  |  |  |  |  |  |  |  |  |
|  | **Collecting duration (min)** | | 1 | 2 | 3 | 4 | 5 | 6 | 7 |

| Table B. Weights of the electret filters before and after collecting human exhale breath particles | **S-6** | **Δm(g)** | **0.001** | **0.003** | **0.004** | **0.005** | **0.004** | **0.005** | **0.005** |
| --- | --- | --- | --- | --- | --- | --- | --- | --- | --- |
|  |  | **After(g)** | 0.272 | 0.275 | 0.312 | 0.352 | 0.346 | 0.366 | 0.317 |
|  |  | **Before(g)** | 0.271 | 0.272 | 0.308 | 0.347 | 0.342 | 0.361 | 0.312 |
|  |  |  |  |  |  |  |  |  |  |
|  | **S-5** | **Δm(g)** | **0** | **0.001** | **0.002** | **0.003** | **0.004** | **0.007** | **0.007** |
|  |  | **After(g)** | 0.271 | 0.312 | 0.286 | 0.282 | 0.311 | 0.334 | 0.341 |
|  |  | **Before(g)** | 0.271 | 0.311 | 0.284 | 0.279 | 0.307 | 0.327 | 0.334 |
|  |  |  |  |  |  |  |  |  |  |
|  | **S-4** | **Δm(g)** | **0.001** | **0.002** | **0.001** | **0.003** | **0.004** | **0.004** | **0.004** |
|  |  | **After(g)** | 0.255 | 0.276 | 0.254 | 0.235 | 0.283 | 0.295 | 0.257 |
|  |  | **Before(g)** | 0.254 | 0.274 | 0.253 | 0.232 | 0.279 | 0.291 | 0.253 |
|  |  |  |  |  |  |  |  |  |  |
|  | **S-3** | **Δm(g)** | **0.002** | **0.005** | **0.007** | **0.001** | **0.014** | **0.024** | **0.014** |
|  |  | **After(g)** | 0.264 | 0.286 | 0.244 | 0.373 | 0.337 | 0.367 | 0.314 |
|  |  | **Before(g)** | 0.262 | 0.281 | 0.237 | 0.372 | 0.323 | 0.343 | 0.3 |
|  |  |  |  |  |  |  |  |  |  |
|  | **S-2** | **Δm(g)** | **0.002** | **0.004** | **0.009** | **0.012** | **0.011** | **0.005** | **0.012** |
|  |  | **After(g)** | 0.286 | 0.308 | 0.373 | 0.348 | 0.385 | 0.3 | 0.312 |
|  |  | **Before(g)** | 0.284 | 0.304 | 0.364 | 0.336 | 0.374 | 0.295 | 0.3 |
|  |  |  |  |  |  |  |  |  |  |
|  | **S-1** | **Δm(g)** | **0** | **0.001** | **0.007** | **0.007** | **0.011** | **0.013** | **0.006** |
|  |  | **After(g)** | 0.229 | 0.237 | 0.299 | 0.226 | 0.29 | 0.292 | 0.25 |
|  |  | **Before(g)** | 0.229 | 0.236 | 0.292 | 0.219 | 0.279 | 0.279 | 0.244 |
|  |  |  |  |  |  |  |  |  |  |
|  | **Breath volume(L)** | | 50 | 100 | 150 | 200 | 250 | 300 | 350 |
